# Clinical Benefit of Second-Line Palliative Chemotherapy in Advanced Soft-Tissue Sarcoma

**DOI:** 10.1155/2010/264360

**Published:** 2010-05-20

**Authors:** Anna Minchom, Robin L. Jones, Cyril Fisher, Omar Al-Muderis, Sue Ashley, Michelle Scurr, Vasilios Karavasilis, Ian R. Judson

**Affiliations:** ^1^Sarcoma Unit, The Royal Marsden Hospital, Fulham Road, London SW3 6JJ, UK; ^2^Fred Hutchinson Cancer Research Center, UW Department of Medicine, Seattle Cancer Care Alliance, Seattle, WA 98109, USA

## Abstract

*Background*. This paper aimed to assess the utility of second-line chemotherapy in patients with advanced soft-tissue sarcoma. *Materials and Methods*. A retrospective search of a prospectively maintained database identified patients treated between 1991 and 2005. Patients with gastrointestinal stromal tumours, small round cell tumours, and Ewing's sarcoma were excluded. Response was assessed using WHO and RECIST. Patients who achieved stable disease for 6 months or more were classified as having disease control. *Results*. Three hundred and seventy-nine patients received second-line chemotherapy. Eighty-six (22.7%) achieved disease control. Median duration of response was 11 months (95% CI: 9–13). On multivariate analysis, pathological subtype, absence of lung metastases, and the use of combination chemotherapy were independent predictors of disease control. Twenty-eight (16.1%) patients who failed to respond to first-line therapy achieved disease control. Eight (2.1%) patients had sufficient downstaging to enable complete surgical resection. Progression-free survival was 23% at 6 months. Median overall survival was 8 months (95% CI: 7–10 months). On multivariate analysis, synovial histology and absence of lung metastases were associated with improved survival. *Conclusion*. Second-line chemotherapy can provide clinical benefit in over 20% of soft-tissue sarcoma patients.

## 1. Introduction

Soft-tissue sarcomas (STSs) are malignant tumors of connective tissue that account for 1% of all human cancers [1]. They consist of approximately 50 different histological subtypes and have differing clinical behavior and response to chemotherapy. The median age at diagnosis is 50 years.

In the advanced disease setting (locally advanced, inoperable or metastatic) palliative chemotherapy is the mainstay of treatment [2], although in a small subset this treatment may be curative [3]. Doxorubicin is, by consensus, the standard first line therapy [4]. Trabectedin has recently emerged as an effective agent in patients who have progressed on an anthracycline and ifosfamide. Trofosfamide and gemcitabine appear to be associated with some activity and tolerability in the doxorubicin/ifosfamide refractory patients [5]. Taxanes, such as paclitaxel have been reported to have activity in vascular sarcomas [5, 6]. There is ongoing work on the potential use of molecular targeted therapies such as antiangiogenic agents, IGF1-R, HDAC, and mTOR inhibitors [7, 8]. There are also ongoing developments in establishing biomarkers to allow further personalisation of therapy [9].

Combination chemotherapy in our practice is largely reserved for those with rapidly progressing metastatic disease or inoperable soft tissue sarcoma where response may render disease resectable. In a recent meta-analysis, three-randomized phase III trials were identified comparing combination chemotherapy regimens containing ifosfamide with regimens without ifosfamide. This analysis revealed that the addition of ifosfamide to a chemotherapy regimen significantly improved response rates but did not produce a significant difference in 1 year survival. Higher rates of adverse events, including myelosuppression and death, were observed in patients who received combination chemotherapy [10].

A retrospective review, performed by the EORTC, of 2185 patients treated with first-line anthracycline-based regimens (within a number of clinical trials) demonstrated an overall one-year survival of 48% and two-year survival of 22%. Good performance status, young age, and absence of liver metastases were associated with improved survival and response to chemotherapy. The poor responsiveness of patients with liver metastases may have been from the inclusion of patients with GIST in older studies. Low histopathological grade was associated with longer survival but a lower response rate. Patients with liposarcoma and synovial sarcoma had significantly longer survival, and those with malignant fibrous histiocytoma had significantly shorter survival. A significantly higher response rate was observed in patients with liposarcoma and a lower response rate in those with leiomyosarcoma [11]. Further review of EORTC data looked specifically at pretreated patients. Progression, free survival at 3 months was 39% for those patients receiving active agents and 21% for those receiving inactive regimens [12]. At six months progression free survival was 14% for those patients receiving active agents and 8% for those receiving inactive regimens. Three prognostic factors were identified: treatment with an active drug, interval since initial diagnosis, and performance status.

A previous study conducted at The Royal Marsden Hospital retrospectively analyzed the efficacy of first-line chemotherapy in a large unselected cohort of patients with advanced STS [13]. The study by Karavasilis demonstrated that approximately 50% of patients benefited from first-line chemotherapy and that synovial sarcoma and liposarcoma were associated with a better prognosis. 

There are, however, no data on the benefit of second-line chemotherapy outside the context of a clinical trial. The aim of this study therefore was to look at a sequential, unselected group of patients being treated as part of routine clinical care in the second-line setting, using the same cohort of patients reported by Karavasilis. The aim was to assess the true clinic benefit derived from second-line chemotherapy for this patient group outside the context of a clinical trial and to delineate potential predictive and prognostic factors in this setting.

## 2. Materials and Methods

Prior to commencing the study, approval was obtained from The Royal Marsden Hospital Audit Committee. A retrospective search of the prospectively maintained The Royal Marsden Hospital Sarcoma Unit database was performed to identify patients registered between 1991 and 2005 who had received second-line chemotherapy for advanced or metastatic disease. Adjuvant chemotherapy was not classified as first-line treatment. Those with osteosarcoma, Ewing's sarcoma, gastrointestinal stromal tumors, rhabdomyosarcoma, and other small round cell tumors were excluded. The analysis only included patients treated with second-line chemotherapy at The Royal Marsden Hospital. All patients had their histology reviewed on referral to The Royal Marsden Hospital. Only those patients with advanced disease (primary tumor or local recurrence not amenable to surgical resection) or metastatic disease were included. Patients who received adjuvant chemotherapy were only included if they subsequently received palliative chemotherapy for recurrent advanced disease.

The following variables were obtained from the database; date of presentation, date of birth, sex, histological subtype, status of disease (advanced/metastatic), sites of metastases at time of second-line chemotherapy, date of second-line chemotherapy, second-line chemotherapy used, and number of cycles administered. Date of last follow-up or death was obtained. The response to treatment was categorized as stable disease (SD), progressive disease (PD), partial response (PR), or complete response (CR). Response to treatment was assessed from radiology reports using WHO or RECIST criteria (more recent reports using RECIST). Duration of response was measured from the first documented radiological evidence until the time of progression. Repeat radiology was performed whilst on chemotherapy every 2 to 3 cycles and whilst not on chemotherapy every 2 to 3 months. Radiological assessment was with cross-sectional imaging or chest radiography in those patients with assessable disease with this modality. Patients were classified as having a disease control if they had CR, PR, or SD for 6 months or more. Use of this classification of response, referred to as “disease control rate” or “progression free rate”, has been proposed as a valid response measurement in a number of tumour types [12, 14].

## 3. Statistical Methods

The following were investigated as potential predictive and/or prognostic factors.

- Age at start of second-line chemotherapy (less than 40 years/40 to 59 years/over 60 years).
- Gender.
- Histology (e.g., leiomyosarcoma/synovial sarcoma/liposarcoma).
- Sites of disease at start of second-line chemo (liver, lung, bone).
- Time from primary diagnosis to second-line chemo (<1 year/1-2 years/>2 years).
- Type of second-line chemotherapy (single agent/combination).

Overall survival was illustrated by means of Kaplan-Meier curves and differences between groups were assessed using univariate analysis by the log-rank test. A multivariate analysis was done to determine the independent significance of variables using the proportional hazards model.

The response rate was expressed as a percentage with 95% confidence interval. 

The influence of prognostic variables on response rate was assessed in a univariate analysis by means of the chi-squared test, Fishers exact test, or the *t*-test and in a multivariate analysis by means of binary logistic regression.

All significance tests were 2-sided and a 5% level of significance was used; no adjustment was made for multiple testing.

## 4. Results

### 4.1. Demographics

Between January 1991 and December 2005, 687 new patients were registered at The Royal Marsden Hospital with locally advanced or metastatic STS. Four hundred and thirty three received second-line chemotherapy. Fifty-four patients were excluded from the analysis; 52 were reviewed at The Royal Marsden Hospital but received second-line chemotherapy at other institutions and 2 were paediatric patients. A total of 379 patients were considered eligible for analysis (176 males and 203 females). The most common histological subtype was leiomyosarcoma (35.4%) followed by synovial (13.7%) and liposarcoma (10.3%). At the start of second-line treatment 40 patients had locally advanced disease (10.6%) and 339 (89.4%) had metastatic disease. Lung was the most common site of metastases (60.9%), followed by liver (19.3%) and bone (6.1%). One hundred and fifty three had metastases in multiple organs (40.4%) (Table 1).

### 4.2. Response to Chemotherapy

Three hundred and twenty six (86.0%) patients were treated with single-agent chemotherapy. Of these 62 received single-agent doxorubicin (19.0%) and 123 (37.7%) single-agent ifosfamide. Thirty-five (10.7%) were treated with a single agent within various Phase 1 trials. One hundred and six patients (28%) received another single agent. Forty-one of these received trabectedin, other regimens included infusional or oral etoposide, paclitaxel, dacarbazine, and gemcitabine. Three patients (0.9%) who had endocrine therapy were also included in the single agent chemotherapy group. Fifty-three patients had combination chemotherapy, 21 with an ifosfamide and doxorubicin combination. A median of 3 cycles of chemotherapy were given, with a range of 1 to 24. No other therapy was given for advanced disease other than pulmonary metastastectomy.

Disease control was defined as CR, PR, or SD of 6 months, or more. If a patient had a CR or PR less than 6 months they were still classified as having achieved disease control (this was the case in 7 patients with a PR). Those with SD for 6 months or more were classified as being progression free for over six months and included in the disease control rate group for analysis. The duration was measured from the first radiological documentation of response. Disease control was seen in 86 (22.7% of patients). Of these 8 (9.3%) had a complete response, 20 (23.3%) had a partial response and 58 (67.4%) had stable disease for 6 months or more. Of the 42 patients receiving trabectedin 12 (28.6%) achieved disease control. Of the 18 patients receiving gemcitabine and docetaxel in combination 4 (22.2%) achieved disease control. Nine (2.4%) of patients were lost to follow up before any radiological assessment was performed and were therefore judged as not assessable (Tables 2 and 3).

**Figure 1:**
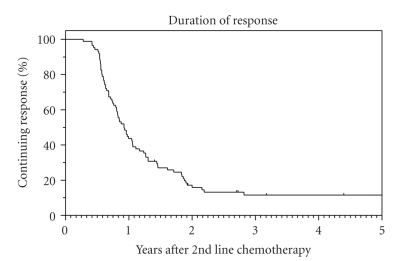
Duration of disease control.

Of those patients who achieved disease control, the duration of control was 11 months (95% CI: 9–13 months). (Figure 1) For patients who achieved stable disease as their best response, the median duration of disease stabilization was 6 months. Fifty-eight of these patients had disease stabilization for 6 months or more.

On univariate analysis, liposarcoma patients had better disease control to second-line chemotherapy than other histological subtypes (*P* = .03). Patients with lung metastases were less likely to achieve disease control (*P* = .04). Patients were more likely to achieve disease control to combination chemotherapy than to single agent chemotherapy (*P* = .02) (Table 4).

Patients who responded (achieved disease control) to first-line chemotherapy were marginally more likely to respond to second-line chemotherapy though this did not reach statistical significance (*P* = .06, Fishers exact test) (Table 5).

On multivariate logistic regression analysis, pathological subtype and lung metastases were independent predictors of disease control to treatment. After adjusting for pathology and lung metastases, patients having combination chemotherapy had a better disease control rate (*P*  =  .01) (Table 4).

### 4.3. Survival Analysis

**Figure 2:**
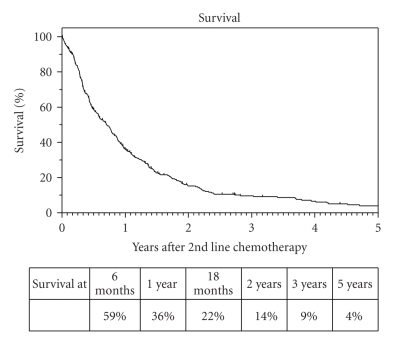
Overall survival.

Progression-free survival was 23% at six months, 11% at one year, and 4% at two years. At the time of analysis 65 patients were alive. Median survival from start of second-line chemotherapy was 8 months (95% CI: 7–10). Thirty six percent of patients were alive at one year and 4% at 5 years (Figure 2).

On univariate analysis, older patients had significantly worse survival (*P* = .03) and those with synovial histology had significantly better survival (*P*  =  .008) (Table 6).

On multivariate analysis, synovial histology (*P*  =  .005) and lack of lung involvement (*P*  =  .01) were significant independent factors associated with improved overall survival. After adjusting for histology and lung disease, patients on combination chemotherapy had better prognosis than those treated with a single agent (*P*  =  .004) in the second-line setting (Table 6).

## 5. Discussion

The role of second-line chemotherapy in metastatic STS is not well established. The aim of our study was to assess response and survival in an unselected cohort of patients with advanced STS treated with second-line chemotherapy at a single centre so providing a reflection of the true benefit derived from second-line chemotherapy in routine practice. Such a study is limited by the availability of retrospectively gathered data and our data do not include primary tumour site or histological grading. However all our patients had advanced progressive disease at the time of treatment so histological grading of the original tumour is of less relevance as a prognostic factor. Patient performance status was also not documented consistently. However, in our clinical practice all patients must have a WHO performance status of 0 to 2 to receive chemotherapy. The study also used a cohort of patients being treated largely in the era before subtype specific therapy for soft tissue sarcoma was practiced so current practice may differ slightly from that practiced in the earlier part of the cohort. 

In our cohort of 379 pretreated patients, 86.0% received single-agent chemotherapy, compared to 61% of patients in our report of first-line therapy [13]. The higher use of combination chemotherapy in the first-line setting is consistent with the use of more toxic combination regimens in potentially resectable disease. Response has been shown to be higher with combination chemotherapy, though with more toxicity [10]. In our study, patients treated with combination regimens (36%) had significantly higher disease control rates than those treated with single-agents (21%). This may be due to the selection of fitter patients for combination chemotherapy. Performance status was not consistently documented in the database and so this important factor could not be included in the analyses. However, no significant difference in median age was observed between those on combination chemotherapy versus single-agent, hence there was no obvious age bias in selection of treatment. Therefore, where tumour shrinkage is the primary goal of treatment or in the case of rapidly progressing disease the use of combination chemotherapy in the second-line setting appears justified.

Patients who respond to first-line chemotherapy were also marginally more likely to respond to second-line chemotherapy, though this did not reach statistical significance. 

The fact that 19% of patients received doxorubicin second-line can be explained by the fact that some patients would have been treated in the context of clinical trials, such as a study comparing ifosfamide with doxorubicin as first-line therapy [15]. Patients with liposarcoma had significantly better response to second-line chemotherapy, and this is consistent with first-line chemotherapy data [11, 13]. Of the 39 liposarcomas, 25 (64.1%) were classified as myxoid/round cell. This tumour subtype has been shown to have better response rates to first-line chemotherapy [16]. The EORTC study also suggested that the absence of liver metastases was associated with a significantly better response to chemotherapy. In contrast, our study suggests that patients with lung metastases were significantly less likely to respond to chemotherapy. However, our analyses were performed by multiple lines of testing, and consequently it is not possible to draw definite conclusions regarding the association between metastatic site and response to second-line chemotherapy. 

Median overall survival in our patients was 8 months, one-year survival 36% and five-year survival only 4%. This is consistent with previous studies estimating median survival to be between 7 and 12 months from commencing first-line chemotherapy [5]. Furthermore, one-year survival has been documented as 48% and five-year survival as 8% in patients treated with first-line chemotherapy [11, 13]. In our study, younger age, synovial histology, and absence of lung metastases were associated with significantly longer survival. Synovial histology has previously been found to be an independent prognostic factor in patients treated with first-line chemotherapy [11, 13].

Survival can be long term in a small subset of patients and an EORTC study has demonstrated that complete remission can occur in all histological subtypes. This was more likely to occur in those who achieved a complete response to first-line chemotherapy [3]. Eight patients in our cohort (2.1%) had sufficient downstaging following second-line chemotherapy to allow surgical resection of residual disease. Four of these patients had synovial sarcoma. 

In conclusion, our study has shown that more than 20% of soft tissue sarcoma patients treated with second-line chemotherapy can obtain prolonged benefit for over 6 months. In addition, confirmed responses were observed in a small proportion of patients who did not respond to first-line chemotherapy. Those with liposarcoma, absence of lung metastases, and patients treated with combination regimens were significantly more likely to respond to second-line therapy. The median duration of response or stable disease was 11 months and in a small subset of patients complete remission was achieved with multimodality management. Our study confirms the differential response of histological subtypes to chemotherapy and the need to select patients for second-line treatment carefully according to those who are more likely to derive clinical benefit. There remains a need for novel effective therapies in metastatic STS, particularly for patients with certain chemoresistant subtypes, who currently have a poor prognosis. 

## Figures and Tables

**Table 1:** Clinical characteristics.

Patients		379

Sex	Male : Female	176 : 203

Age at 2nd-Line chemo (years)	Median (Range)	50 (18–81)
Histology	Leiomyosarcoma	134 (35.4%)
	Synovial sarcoma	52 (13.7%)
	Liposarcoma	39(10.3%)
	Other:	154 (40.6%)
	Malignant Fibrous Histiocytoma	25
	Sarcoma (not otherwise specified)	30
	Fibrosarcoma	9
	Malignant Peripheral nerve sheath tumour	12
	Angiosarcoma	18
	
	Other	60

Disease	Advanced	40 (10.6%)
	Metastatic—single organ	186 (49.1%)
	Metastatic—multiple organs	153 (40.4%)

Sites of metastases	Lung	231 (60.9%)
	Liver	73 (19.3%)
	Bone	23 (6.1%)

**Table 2:** Second-line chemotherapy regimens and disease control rate.

Chemotherapy	Single agent doxorubicin					62 (16.4%)	
	Single agent ifosfamide					123 (32.5%)	
	Other Single agent					106 (28.0%)	
	Doxorubicin/ifosfamide					21 (5.5%)	
	Other combination					32 (8.4%)	
	Phase I trial					35 (9.2%)	

Number of Cycles	1	2	3	4	5	6	>6
	72	111	35	49	16	68	28

Response	CR					8 (2.1%)	
	PR					20 (5.3%)	
	SD					76 (20.1%)	
	PD					266 (70.2%)	
	NA					9 (2.4%)	

Disease control rate	CR/PR/SD >6 months					86 (23.2%)	
	Non responder					284 (76.8%)	

CR: complete response, PR: partial response, SD: stable disease, PD: progressive disease, NA: not assessable.

**Table 3:** Response to second-line chemotherapy according to pathology and regime.

Histology	Chemo	CR	PR	SD	PD	NA
Synovial	Dox		1 (0.3%)		3 (0.8%)	1 (0.3%)
	Ifos	1 (0.3%)	4 (1.1%)	2 (0.5%)	15 (4.0%)	1 (0.3%)
	Dox/Ifos	1 (0.3%)	2 (0.5%)	1 (0.3%)	2 (0.5%)	
	Other	2 (0.5%)		4 (1.1%)	13 (3.4%)	

Liposarcoma	Dox	1 (0.3%)	1 (0.3%)	3 (0.8%)	2 (0.5%)	
	Ifos		2 (0.5%)	3 (0.8%)	14 (3.7%)	
	Dox/Ifos			5 (1.3%)	1 (0.3%)	
	Other		1 (0.3%)	5 (1.3%)	6 (1.6%)	

Leiomyosarcoma	Dox		1 (0.3%)	7 (1.8%)	12 (3.2%)	1 (0.3%)
	Ifos		1 (0.3%)	4 (1.1%)	27 (7.1%)	2 (0.5%)
	Dox/Ifos		1 (0.3%)		2 (0.5%)	
	Other		2 (0.5%)	14 (3.7%)	59 (15.6%)	

Other:	Dox			6 (1.6%)	23 (6.1%)	
	Ifos	1 (0.3%)	2 (0.5%)	11 (2.9%)	31 (8.2%)	2 (0.5%)
	Dox/Ifos				6 (1.6%)	
	Other	2 (0.5%)	2 (0.5%)	11 (2.9%)	50 (13.2%)	2 (0.5%)

Dox: doxorubicin, Ifos: Ifosfamide, CR: complete response, PR: partial response, SD: stable disease, PD: progressive disease, NA: not assessable.

**Table 4:** Univariate and multivariate analysis of disease control rate.

Univariate analysis			

		Disease control rate	

All Patients		23.2% (95% CI:18.9%–27.5%)	
Sex	Male	24%	*P* = .6
	Female	22%	

Age (years)	0–39	24%	*P* = .2
	40–59	27%	
	60+	16%	

Histology	Leiomyosarcoma	19%	*P* = .2
	Synovial sarcoma	34%	*P* = .07
	Liposarcoma	38%	*P* = .03
	Other	19%	

Disease	Locally advanced	18%	*P* = .1
	Metastatic—single organ	29%	
	Metastatic—multiple organs	17%	

Sites of metastases	Involved versus uninvolved		
	Lung	19% versus 29%	*P* = .04
	Liver	27% versus 22%	*P* = .4
	Bone	23% versus 23%	*P* = 1.0

Chemotherapy	Single agent/Phase I	21%	*P* = .02
	Combination	36%	

Chemo Regimen	SA Doxorubicin	27%	
	SA Ifosfamide	21%	
	Other SA	22%	
	Phase I trial	9%	
	Dox/Ifos combination	38%	
	Other combination	34%	

Multivariate analysis			

	Relative likelihood of disease control (95% CI)		Significance

Pathology type			
Liposarcoma versus other	2.5	1.2–5.1	*P* = .02
Synovial versus other	2.3	1.2–4.5	*P* = .02

Sites of disease			
Lungs Involved	0.6	0.3–1.0	*P* = .04

Not involved	1.0
Chemotherapy			
Single Agent/Phase 1 Combination	1.0	1.2–4.4	*P* = .01
	2.3		

SA: single agent, Dox/ Ifos: Doxorubicin/Ifosfamide.

**Table 5:** Disease control rate of first-line chemotherapy against disease control rate of second-line chemotherapy.

		1st-line chemotherapy	
		Responder	Non responder
2nd-line chemotherapy	Responder	42	28
	Non responder	130	145

**Table 6:** Univariate analysis (Life table & Logrank) and multivariate analysis (Cox regression) of survival.

Univariate analysis			

		Median Survival (months)	Significance
Sex	Male	8	*P* = .4
	Female	9	

Age	0–39	9	*P* = .03
	40–59	9	
	60+	7	

Histology	Leiomyosarcoma	9	Synovial versus the rest *P* = .008
	Synovial	12	
	Liposarcoma	11	
	Other	7	

Disease	Locally Advanced	8	*P* = .3
	Metastatic—single organ	9	
	Metastatic—multiple organs	8	

Sites of metastases	Involved versus uninvolved		
	Lung	7 versus 10	*P* = .2
	Liver	8 versus 8	*P* = .5
	Bone	12 versus 8	*P* = .7

Multivariate analysis			

	Relative risk	95% CI	Significance

Histology			
Synovial	0.6	0.5–0.9	*P* = .005
Other histology	1.0		

Site			
Not involved	1.0	1.1–1.7	*P* = .01
Involved	1.4		

Chemotherapy			
Combination	0.6	0.4–0.9	*P* = .004
Single agent/Phase I	1.0		
